# Machine learning integrated clinical-proteomics data identifies a 6-protein panel signature for atherosclerotic severity and enhanced patient stratification

**DOI:** 10.1186/s43556-026-00438-z

**Published:** 2026-04-10

**Authors:** Mª Jesús Extremera-García, Marta Rojas-Torres, Blanca Priego-Torres, Lucía Beltrán-Camacho, Sara Eslava-Alcón, Francisco Rodríguez-Martín, Josefa Benítez-Camacho, Antonio Ballesteros-Ribelles, Ana Martínez del Val, Jesper Olsen, Eva Lozano-Loaiza, Mª Ángela González-García, Daniel Sanchez-Morillo, Alejandro Fernández-Vega, Joan Montaner, Esther Doiz, Manuel Rodriguez-Piñero, Mª Carmen Durán-Ruiz

**Affiliations:** 1 Biomedicine, Biotechnology and Public Health Department, Cádiz University, Cádiz, Spain//Biomedical Research and Innovation Institute of Cadiz (INiBICA), Cadiz, Spain; 2 UGC Laboratory Medicine, University Hospital Puerta del Mar, Cádiz, Spain; 3 Automation Engineering, Electronics and Computer Architecture and Networks Department, University of Cádiz, Cádiz, Spain//Biomedical Research and Innovation Institute of Cadiz (INiBICA), Cadiz, Spain; 4 Institute of Biomedicine of Seville (IBIS), Virgen del Rocio University Hospital//CSIC/Dpt. Cell Biology, Faculty of Biology, University of Seville, Seville, Spain; 5 National Center of Cardiovascular Research Carlos III (CNIC), Madrid, Spain; 6 Novo Nordisk Foundation Center for Protein Research, University of Copenhagen, Copenhagen, Denmark; 7 Internal Medicine Unit, La Línea Hospital, La Línea de La Concepción, Cádiz, Spain; 8 UGC Laboratory Medicine, University Hospital of Jerez de La Frontera, Cádiz, Spain; 9 Neurovascular Research Group, Institute de Biomedicine of Seville, IBiS/Virgen, Macarena University Hospital/CSIC/University of Seville, Seville, Spain; 10 Angiology and Vascular Surgery Unit, University Hospital Puerta del Mar, Cádiz, Spain//Biomedical Research and Innovation Institute of Cadiz (INiBICA), Cadiz, Spain

**Keywords:** Atherosclerosis, Dyslipidemia, Serum biomarkers, Machine learning, Precision medicine, Proteomics

## Abstract

Atherosclerosis, a major cause of adverse cardiovascular events and mortality rates worldwide, stems from sustained lipid accumulation and subsequent chronic inflammation within the arterial walls. An early identification of patients at risk is crucial to prevent life-threatening thrombotic events and provide effective and personalized treatments. Leveraging the power of machine learning (ML) to enhance diagnostics and biomarker discovery, we applied a high-throughput approach using five ML classification algorithms (MLCA), integrating clinical and serum proteomic data from patients with carotid atherosclerotic stenosis (AT, n:60), dyslipidemic patients (DLP, n:55), and healthy controls (HC, n:66). As a result, a robust 6-protein panel (B2M, GPV, MMP9, PLF4, TSP1, and FB isoforms) was identified with a ROC-AUC value > 0.9 for all algorithms applied, highly discriminating AT patients compared to DLP or CTRL. The levels of these proteins were further validated in an independent external cohort, including patients presenting with acute atherothrombotic stroke, corroborating the potential of this panel as biomarker for atherosclerosis severity. In addition, the combined clinical-proteomic ML approach provided a more accurate patient stratification than the clinical or proteomic analysis alone. Mechanistically, the identified biomarkers highlight the importance of platelet activation, uncontrolled angiogenesis and intraplaque haemorrhage in the atherosclerotic process, underscoring the need for *multipathway* therapies to prevent unwanted thrombotic events.

**Supplementary Information:**

The online version contains supplementary material available at 10.1186/s43556-026-00438-z.

## Introduction

Atherosclerotic cardiovascular diseases (ACVD)—including coronary artery disease, stroke and peripheral artery disease- are a major cause of high mortality rates worldwide. The common underlying factor is atherosclerosis, a multifactorial process initiated by the accumulation of lipids and fibrous material in the artery walls [1]. This is followed by oxidation and aggregation of the pro-atherogenic lipidic particles which promote an inflammatory response, endothelial damage, vascular fibrosis and, ultimately, the formation of the so-called atherosclerotic plaques (AP) [2]. While advanced atheroma plaques can significantly reduce the arterial lumen, causing blood flow reduction and ischemia, the rupture of unstable plaques triggers thrombotic events, leading to myocardial infarction, or stroke [3]. Thus, identifying markers linked to atherosclerosis progression constitutes a major research focus, both to understand the molecular mechanisms underlying AP formation and to predict the risk of plaque rupture and severe fatal events.

While imaging strategies can identify structural indicators of prone-rupture AP [4], these tools are not always affordable or available, and they require an expert interpretation [5, 6]. Alternatively, the identification of biological markers has been explored, primarily in serum [7], but also in other body fluids like urine or the AP secretome itself [8]. Given the primary role of dyslipidemia in ACVD [9], the "cholesterol hypothesis" has been a cornerstone of cardiovascular (CV) prevention and treatment for decades. Traditional calculators such as Framingham Risk Score and the ACC/AHA Pooled Cohort Equations, consider serum lipid profiles alongside other CV risk factors—such as age, sex, smoking, obesity or diabetes- to stratify an individual´s risk of experiencing a CV event in the next 10 years [10, 11]. However, despite being highly validated, the accuracy of some screening methods remains limited as they do not adequately capture risk stratification in subclinical atherosclerosis [12, 13]. Furthermore, notwithstanding the success of lipid-lowering strategies (e.g., statins, PCSK9 inhibitors, ezetimibe, etc.) [14, 15], the residual risk of ACVD and adverse CV events remains high [16, 17]. Consequently, CV risk estimation focused primarily on LDL-c appears insufficient [18], highlighting the need to incorporate additional markers representative of other causal pathways, such as inflammation or metabolic dysfunction, to fully address the complexity of the atherosclerotic phenomenon [19–21].

In this context, the inclusion of high-throughput omic techniques, in particular genomics and proteomics, has revolutionized modern medicine. Omic approaches, coupled with ML tools now exploiting the vast dataset to its fullest potential, have allowed researchers to reach a more complete molecular picture of the pathophysiology, while also enabling optimized risk stratification [22]. Currently, different studies highlight the benefits of using the full proteomic data to predict CVD risk, including the fact that they can reduce the number of false negatives [23]. Moreover, ML offers the ability to handle non-linear relationships and high-dimensional data far beyond the capacity of traditional statistical models. These tools hold great potential to overcome the limitations of current stratification approaches, thereby enabling the design of personalized strategies for disease prevention and treatment. Ultimately, this guides biomedical research, and cardiovascular medicine in particular, into a new era of precision medicine [24, 25].

With the aim of identifying markers beyond the classical cholesterol metrics, and providing an optimized panel for stratifying severe atherosclerosis, in the current study we applied a multidimensional approach, involving five MLCA, to analyze clinical and serum proteomic data from dyslipidemic patients (DLP), advanced carotid stenosis patients (AT), and healthy donors (CTRL). The integrated strategy, combining clinical and proteomic data, outperformed single-data analyses, yielding a panel of highly discriminant serum markers that may serve as a signature of atherosclerotic severity.

## Results

### Characteristics of the study population

**Fig. 1:**
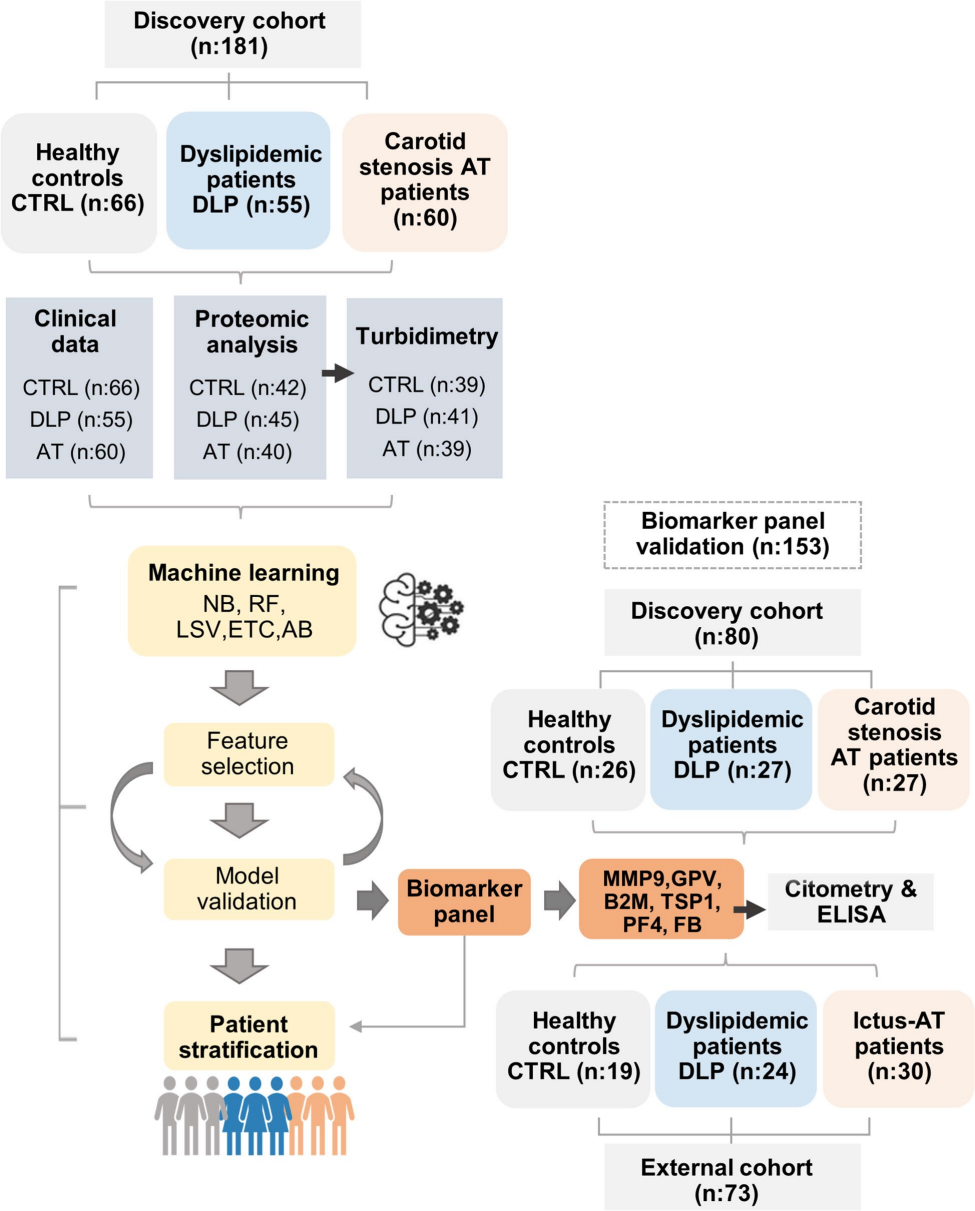
Workflow of the cohort analysis performed in discovery and validation datasets. Clinical parameters, proteomic analysis and turbidimetric validation data registered during the discovery phase were further analyzed by MLCA, obtaining selected features and patient stratification. Next, the biomarker panel was further validated by flow cytometry and ELISA with individuals from the discovery and external independent cohorts. The total number of individuals per group as well as the number of samples used per assay is shown

A total of 254 participants were enrolled in this study, comprising a discovery cohort (n: 181) in which clinical and proteomic data were collected, and an external cohort (n:73) for biomarker validation. An overview of the study workflow, including the number of participants in the assays performed at each stage, is shown in Fig. 1. To evaluate the characteristics of our study population, demographic features, clinical information and biochemical indicators were recorded at the time of sample collection for the three groups evaluated at the discovery phase (CRTL; DLP and AT patients) (Table 1, Table S1). According to these data, the percentage of female (F) was similar in CRTL (50%) and DLP (51%) groups, but decreased drastically in AT (15% F) (Table 1). Besides, CRTL individuals were 10–15 years younger, on average, than DLP or AT patients. The atherosclerotic group displayed the highest prevalence of associated risk factors, including hypertension (HTA, 68%) and diabetes (48%), compared to DLP (HTA, 41% and DM, 17%) and CTRL (HTA, 10% and DM 0%). Also, AT patients reported the highest usage of lipid lowering (68%), antidiabetic (34%) or antihypertensive (51%) drugs, alongside antiplatelet treatments (aspirin, 70%; clopidogrel intake, 30%). Regarding the lipid profile (total cholesterol (TCol), LCL-c, HDLC, Triglycerides (TG)), most lipid features were upregulated in DLP patients compared to CTRL, as expected, but also regarding AT, likely because AT patients were on long-term intake of lipid-lowering therapy (Table 1). Conversely, other CV risk factors (glucose, HbA1c, CRP, etc.) were statistically higher in AT than in CTRL and DLP patients, whereas no significant differences were observed between these two groups. Overall, while DLP displayed the most altered lipid values, the AT group exhibited the poorest clinical and biochemical profile, with unfavorable values for CV risk factors, inflammatory markers and other relevant parameters associated with ACVD, despite pharmacologically lowered lipid levels.

**Table 1:** Baseline characteristics registered for the entire discovery phase cohort. Statistical differences were calculated with Chi square test or Kruskal–Wallis and Dunn´s as post hoc tests. Significance was established at *p*-value ≤ 0.05 (*bolded)

	**CTRL**		**DLP**		**AT**					
	**n**	**n (%) or Mean ± sd**	**n**	**n (%) or Mean ± sd**	**n**	**n (%) or Mean ± sd**	***P* value**	***p* value (DLP *vs* CTRL)**	***p* value (AT *vs* CTRL)**	***p* value (AT *vs* DLP)**
Demographic features										
Sex (female%)	66	33 (50%)	55	28 (51%)	60	9 (15%)	< 0.001	0.921	< 0.001	< 0.001
Age (years)	66	49.80 ± 13.80	55	58.76 ± 12.66	60	64.00 ± 8.75	< 0.001	0.001	< 0.001	0.066
CV Risk factors										
DM (%)	21	0 (0%)	54	9 (17%)	59	28 (48%)	< 0.001	0.054	< 0.001	< 0.001
Hypertension (%)	21	2 (10%)	54	22 (41%)	59	40 (68%)	< 0.001	0.012	< 0.001	0.004
Dyslipidemia (%)	21	0 (0%)	54	52 (96%)	59	43 (73%)	< 0.001	< 0.001	< 0.001	< 0.001
Smoking (%)	33	3 (9%)	47	7 (15%)	55	41 (75%)	< 0.001	0.512	< 0.001	< 0.001
Alcohol consumption (%)	33	0 (0%)	47	0 (0%)	56	7 (13%)	0.005	NA	0.043	0.015
Lipid profile										
TChol (mg/dL)	65	182.06 ± 28.49	55	222.18 ± 41.81	50	177.00 ± 53.48	< 0.001	< 0.001	0.924	< 0.001
HDLc (mg/dL)	64	56.73 ± 11.60	55	53.40 ± 15.49	45	42.49 ± 10.73	< 0.001	0.144	< 0.001	< 0.001
LDLc (mg/dL)	63	111.41 ± 26.87	55	141.85 ± 42.82	45	110.62 ± 50.34	< 0.001	< 0.001	1	< 0.001
No HDLc	64	125.09 ± 26.62	55	168.78 ± 46.44	50	139.64 ± 50.48	< 0.001	< 0.001	0.417	0.001
TG (mg/dL)	65	80.37 ± 32.80	55	156.09 ± 102.05	50	150.32 ± 72.98	< 0.001	< 0.001	< 0.001	1
VLDLc calc	65	16.074 ± 6.56	55	31.22 ± 20.41	50	30.06 ± 14.6	< 0.001	< 0.001	< 0.001	1
Other CV related parameters										
Glucose (mg/dL)	66	90.39 ± 10.61	55	99.45 ± 20.23	59	127.02 ± 58.79	< 0.001	0.089	< 0.001	0.162
HbA1c (%)	46	5.391 ± 0.35	55	5.633 ± 0.67	24	6.671 ± 1.45	0.001	0.357	< 0.001	0.019
CRP (mg/L)	51	2.298 ± 2.17	53	3.519 ± 4.84	31	19.458 ± 39.30	< 0.001	1	< 0.001	0.001
Hemogram										
Erythrocytes (count) × 10e^6^/mL	65	4.70 ± 0.51	48	4.82 ± 0.4	58	4.54 ± 0.49	0.021	0.373	0.499	0.016
Hemoglobin (g/dL)	65	14.06 ± 1.27	48	14.75 ± 4.19	58	13.58 ± 1.67	0.229	NA	NA	NA
Platelets (count) × 10e^3^/mL	65	240.69 ± 56.26	48	241.96 ± 66.83	58	240.24 ± 96.61	0.797	NA	NA	NA
Red cell distribution width (volume) %	65	12.971 ± 0.87	48	13.69 ± 1.28	38	24.06 ± 15.11	< 0.001	0.001	< 0.001	0.015
Leucocytes (count) × 10e^3^/mL	65	6.344 ± 1.62	48	7.05 ± 2.00	58	9.11 ± 2.93	< 0.001	0.186	< 0.001	< 0.001
Neutrophils (count) × 10e^3^/mL	65	3.62 ± 1.35	48	3.68 ± 1.34	58	5.72 ± 2.50	< 0.001	1	< 0.001	< 0.001
Treatments										
Antidiabetic drugs (%)	33	0 (0%)	53	10 (19%)	59	20 (34%)	0.001	0.012	< 0.001	0.073
Antihipertensive drugs(%)	33	7 (21%)	53	23 (43%)	59	30 (51%)	0.02	0.036	0.005	0.43
Lipid lowering therapy (%)	33	3 (9%)	53	30 (57%)	59	40 (68%)	< 0.001	< 0.001	< 0.001	0.222
Aspirin intake (%)	33	0 (0%)	55	11 (20%)	60	42 (70%)	< 0.001	0.002	< 0.001	< 0.001
Clopidogrel intake (%)	33	0 (0%)	55	1 (2%)	60	18 (30%)	< 0.001	1	< 0.001	< 0.001
Adverse Drug reactions (%)	66	0 (0%)	55	1 (2%)	60	10 (17%)	< 0.001	0.455	< 0.001	0.009

*n* Number of individuals included per variable, *CTRL* Healthy controls, *DLP* Dyslipidemic patients, *AT* Atherosclerotic patients, *DM* diabetes mellitus, *ADR* adverse drug reactions, *TCol* total cholesterol, *HDLc* high density lipoprotein cholesterol, *LDLc* low density lipoprotein cholesterol, *TG* triglycerides, *CRP* C-reactive protein, *HbA1c* Hemoglobin A1c

### Atherosclerotic patients display a differential proteomic profile

**Fig. 2:**
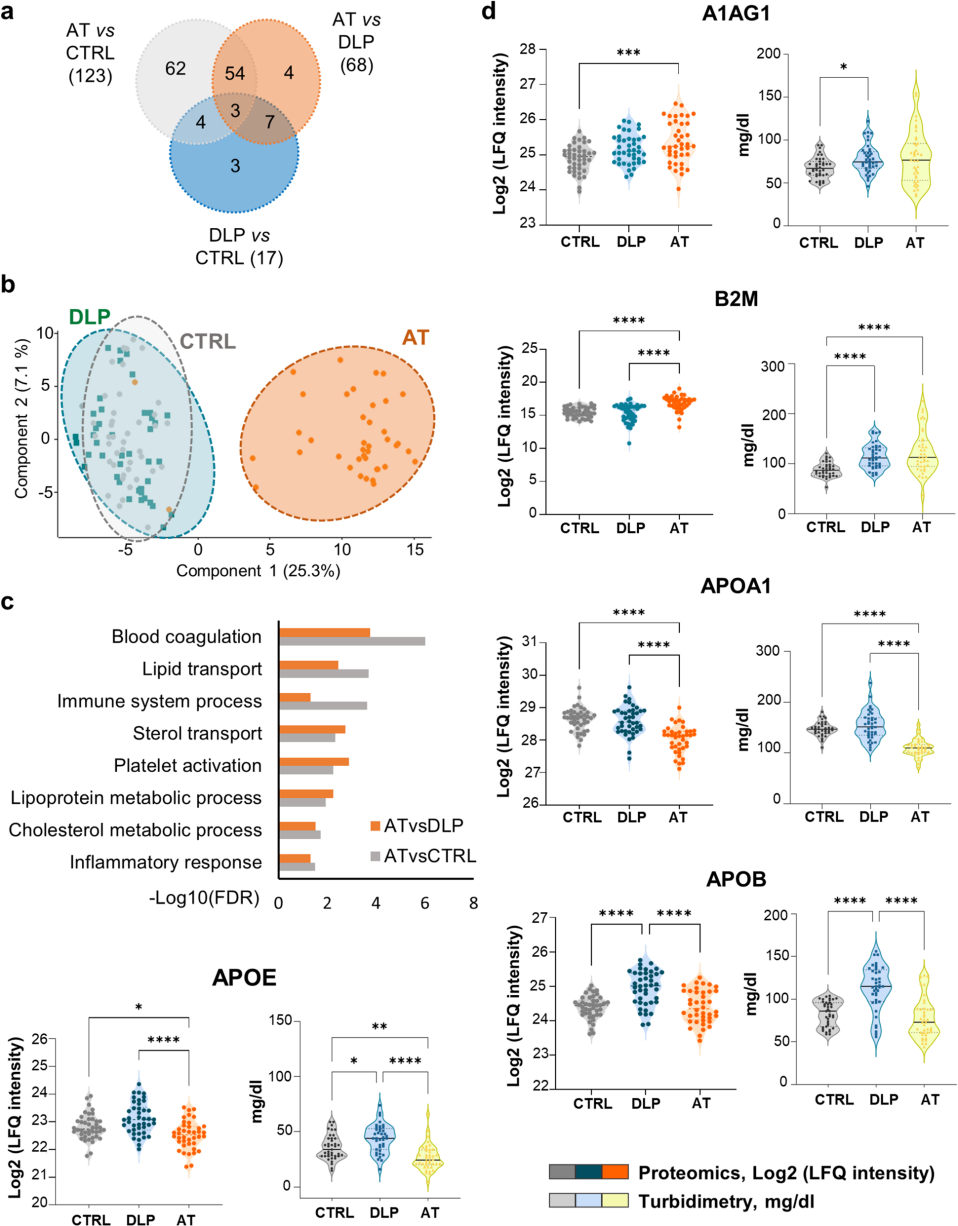
Proteomics revealed a differential protein profile in AT patients. **a** Venn diagrams indicate common and exclusive proteins identified between AT *vs* CTRL, AT *vs* DLP, or DLP *vs* CTRL. **b** Representative PCA plot demonstrating that, based on their proteomic profiles, the AT group is clearly separated from both DLP and/or CTRL individuals. **c** Significant biological functions of the differential proteins identified, ranked by the -log10 (FDR), provided by String platform. **d** Violin plots for selected atherosclerosis-related markers are shown (median, min and max values), representing proteomic changes (Log2 LFQ-Intensity) and corresponding validation by turbidimetry (mg/dl). Statistical differences were calculated with Kruskal Wallis test and Dunn´s as post hoc test: *p-value < 0.05, **p-value < 0.01, ***p-value < 0.001

In total, 304 proteins were identified by label free quantitative (LFQ) proteomic analysis of serum samples from CTRL (n: 42), DLP (n: 45) and AT (n: 40) patients (Table S2). Most protein changes were found in AT *vs* CTRL (123 proteins) and between AT *vs* DLP groups (68), while DLP *vs* CTRL groups yielded only 17 differential proteins (Fig. 2a). Of these, 54 protein changes were common between AT *vs* CTRL and AT *vs* DLP, and only 3 proteins were consistently altered across all three comparisons. Principal component analysis (PCA, Fig. 2b) corroborated that the AT serum proteome profile was distinct from DLP and CTRL groups, whereas the latter two were barely distinguishable. Besides, according to the functional enrichment analysis performed with the plasma proteome as background database, the differential proteins identified were primarily involved in blood coagulation, lipid transport/metabolism and immune response (Fig. 2c), many of which are closely linked to ACVD (Table S3).

Given the known association of several identified proteins (APOB, APOAI, APOE, A1AG1, B2M) with atherosclerosis, their levels were subsequently quantified by turbidimetry (Fig. 2d, Table S4). Turbidimetric results corroborated proteomic data, revealing a significant increase of acute phase α1-acid glycoprotein (A1AG1) and β2-microglobulin (B2M) in the serum of AT and DLP patients, respectively, compared to CTRL. Conversely, Apolipoproteins ApoA1, ApoB and ApoE were significantly downregulated in AT compared to DLP patients and CTRL (Fig. 2d). As expected, these apolipoproteins showed strong correlations with cholesterol-related molecules (LDLc, HDLc, etc.) within the DLP and AT groups (Tables S5 and S6). Finally, because these five proteins can be readily quantified by turbidimetry alongside routine clinical parameters, their measurements were incorporated into the clinical dataset for further ML analysis.

In summary, the AT protein profile was distinct from both the DLP and CTRL groups, whereas few differential proteins were found between the latter two cohorts. Most differential proteins were primarily linked to ACSV related-processes (e.g. lipid metabolism, inflammation).

### Machine learning integration of clinical and proteomic data provides enhanced predictive performance and feature selection

**Fig. 3:**
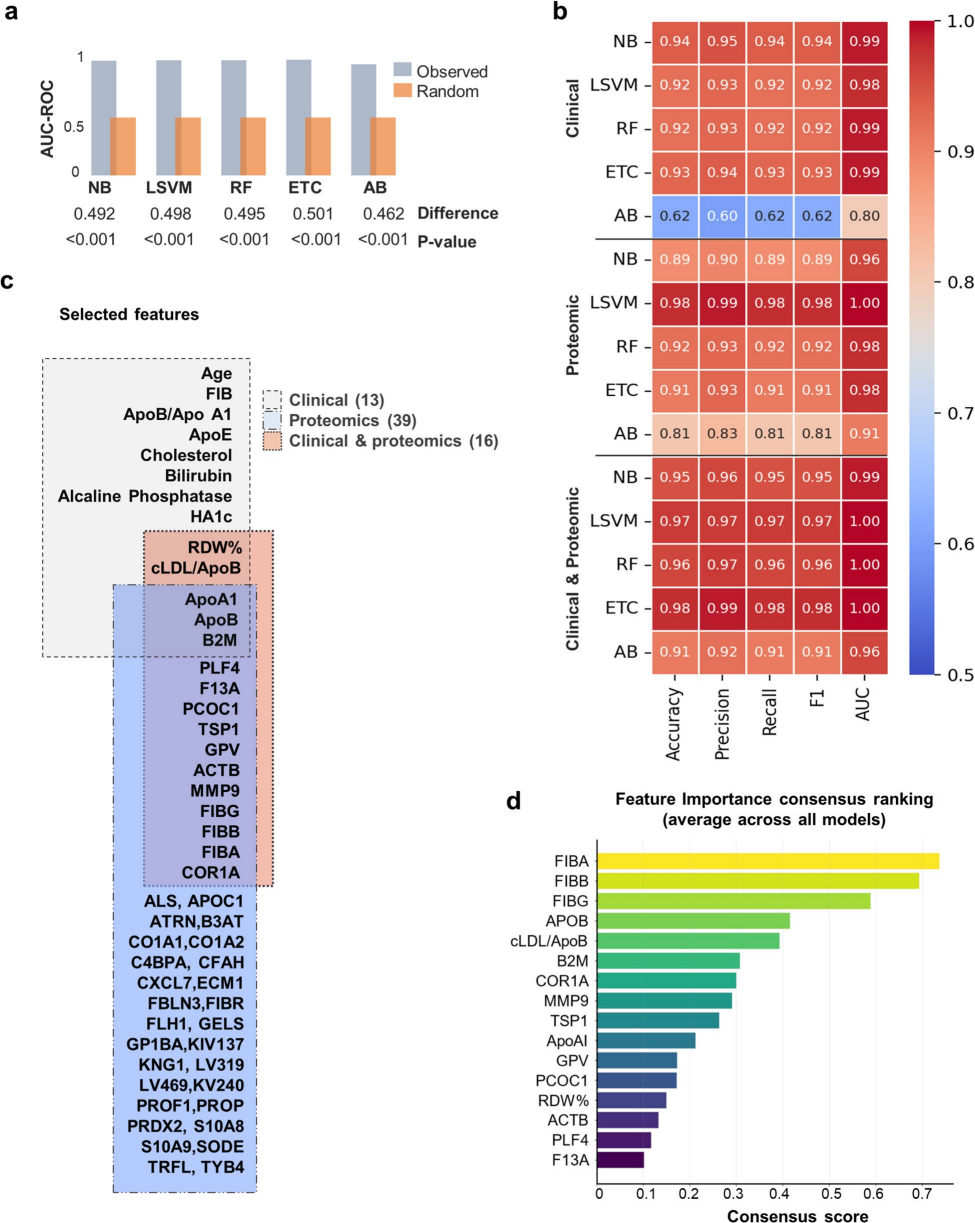
Machine learning training process and feature selection. **a** Permutation tests (1,000 iterations) results indicate that AUC-ROC values (observed) for all 5 MLCA models (NB, LSVM, RF, ETC, AB) were significantly higher (*p*-value < 0.001) than random chance (mean random AUC ≈ 0.5). **b** Performance metrics (Accuracy, macro-averaged Precision, micro-averaged Recall, F1-score, AUC-ROC) for the clinical, proteomic, and combined clinical& proteomic approach. **c** Features selected, from the discovery phase, by the 5 MLCA consensus strategy for the clinical-only, proteomic-only, and combined clinical-proteomic approach. **d** Feature importance consensus ranking obtained across all 5 models

Five different MLCA were applied- Naive Bayes (NB) [26], Linear SVM (LSVM) [27], Random Forest (RF) [28], Extra Trees Classifier (ETC) [29], AdaBoost (AB) [30])—to evaluate individual or integrated proteomic and clinical data from the discovery cohort (Fig. 3). Briefly, the clinical dataset included demographic variables (age or sex percentages), 49 biochemical and hemogram features, and the turbidimetric results of selected proteins (APOB, APOA1, APOE, B2M, A1AG1). Regarding the proteomic data, all 304 proteins identified by MS were considered. The full list of parameters selected are shown in in Table S7.

Separate and integrated clinical-proteomic datasets underwent rigorous processing to identify robust feature predictors while minimizing information leakage. Specifically, a 3-repeated, fivefold stratified cross-validation and internal bootstrap resampling was applied. In each bootstrap run, variables were standardized solely on the training data and then evaluated with the MLCA models (NB, LSVM, RF, ETC, AB). In addition, permutation tests (1,000 iterations) confirmed that all 5 classifiers significantly outperformed random label shuffling (*p* < 0.001; mean random AUC ≈ 0.5) (Fig. 3a), demonstrating that these models captured genuine signal rather than random noise, despite the limited cohort size. Nevertheless, further validation in larger, independent datasets is warranted.

Following consensus-based feature selection, the five classifiers achieved high and consistent predictive performance across all metrics (Fig. 3b). Notably, the integrated clinical-proteomic strategy consistently outperformed individual modalities, reporting the best predictive performance across all classifiers, with ETC, RF and LSVM models achieving AUC-ROC values of 1 and 95% CI between 0.976–0.999 in most cases (Fig. 3b, Table S8). Overall, these findings demonstrate the robustness of the MLCA models applied within our study cohort, and confirm that integrating clinical and proteomic data enhances discriminative power beyond either modality alone.

Regarding the features selected by MLCA (Fig. 3c), the clinical-only analysis identified 13 highly discriminating features across groups, including age, HbA1C, alkaline phosphatase, red blood cell distribution width (RDW %), cholesterol, and several lipid-related proteins and inflammatory markers previously identified by proteomics (ApoB, ApoAI, ApoE, B2M). The proteomic-only analysis identified 39 discriminating proteins, also including APOB, APOA1, fibrinogen and B2M. The integration of both datasets significantly refined this selection to a final 16-features panel, which included specific clinical features (ApoAI, ApoB, LDL cholesterol to ApoB ratio, RDW *%*), and 10 additional proteomic markers: ACTB, COR1A, FIBA, FIBB, FIBG, GPV, MMP9, PCOC1, PLF4, B2M and TSP1. The consensus feature importance ranking for the combined clinical-proteomic dataset is shown in Fig. 3d.

### Identification of a panel biomarker for atherosclerosis severity

**Fig. 4:**
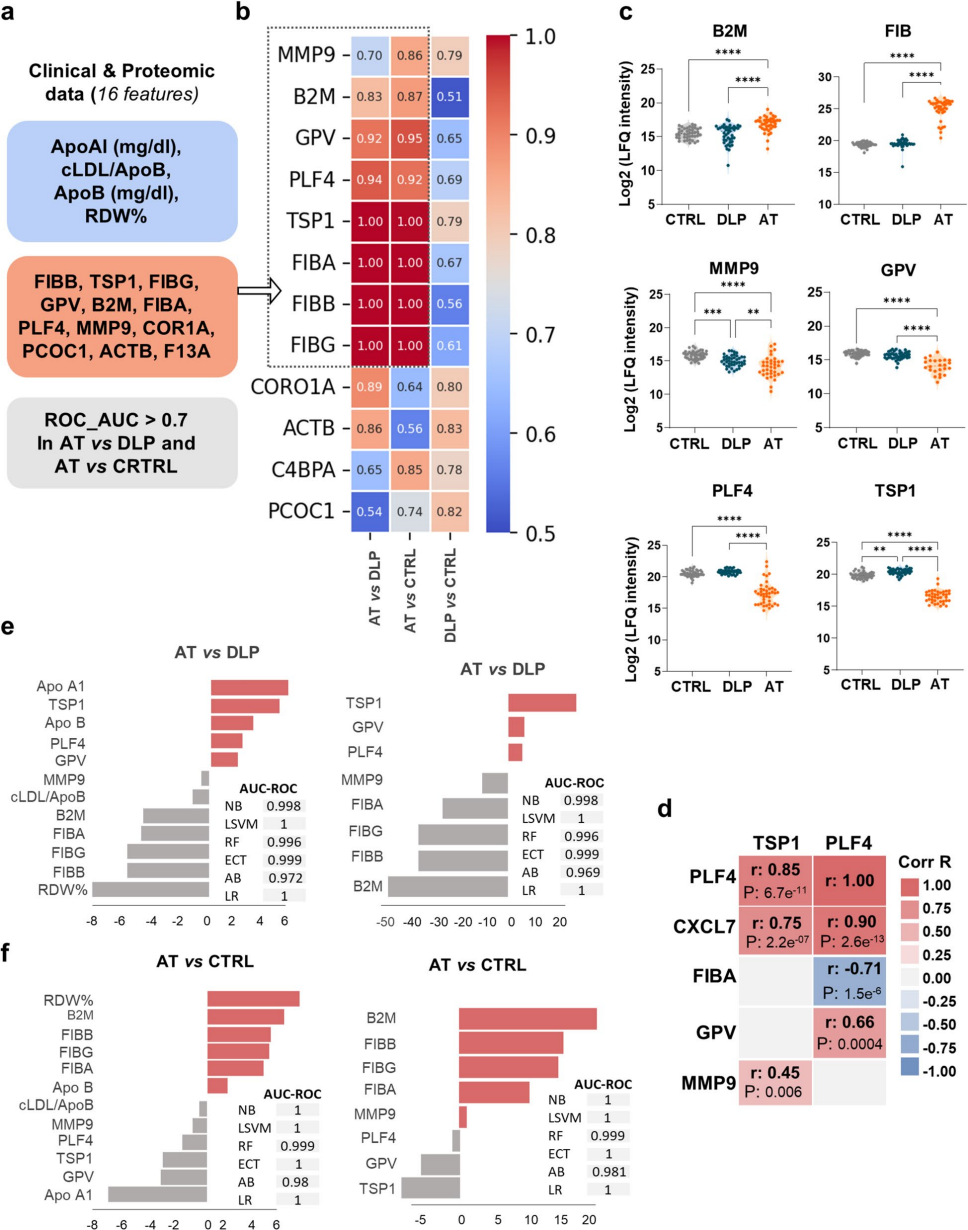
Proteins highlighted by MLCA. **a** MLCA of proteomic&clinical data identified 16 discriminating features among CTRL, DLP and AT patients, including lipid related markers, RDW% and 10 proteomic features. **b** Following an additional filtering step, only those proteins discriminating the AT group from CTRL and DLP (AUC-ROC values > 0.7) were retained for further analysis. **c** Representative violin plots (median, min, max) showing proteomic changes (log2 LFQ intensity) for the 6-protein panel across the study groups. Significant differences were calculated with Kruskal Wallis test and Dunn´s as post hoc test (**p-value < 0.01, ***p-value < 0.001, ****p-value < 0.0001). **d** Representative correlations (Pearson´s r and *p*-values) found in AT patients between selected serum proteins. **e** Nomogram-like coefficient plots and AUC-ROC values for the 5 MLCA classifiers and random classification (LR), comparing the results for the entire set of lipid-related markers and the 6-protein panel, against the 6-proteomic subset alone in the comparisons AT vs DLP and in (**f**) AT vs CTRL

Given the superior performance of the combined clinical-proteomic approach, the 16-feature panel was further filtered, to provide a refined serum marker for atherosclerosis severity (Fig. 4a). Specifically, only those proteomic markers with AUC-ROC values > 0.7 in AT serum compared to both, DLP and CTRL groups (AT *vs* DLP and AT *vs* CTRL) were retained: MMP9, B2M, GPV, PLF4, TSP1, and FIB (in its 3 subunits, FIBA, FIBB, FIBG) (Fig. 4b).

Based on the LFQ proteomic analysis, the inflammatory proteins B2M and Fibrinogen were significantly upregulated in AT patients compared to CTRL and DLP individuals, whereas Platelet Glycoprotein V (GPV), Metalloproteinase-9 (MMP9), platelet factor-4 (PLF4) and Thrombospondin-1 (TSP1) were all downregulated in AT patients (Fig. 4c). Notably, strong correlations were observed among these proteins, particularly within the AT group, but also in the DLP cohort (Tables S5-S6; representative correlations in Fig. 4d).

Collectively, the 6-protein panel achieved AUC-ROC values > 0.97 across all 5 models (Fig. 4e-f), outperforming the results obtained for individual proteins as well as the clinical features alone (ApoA1, ApoB, cLDL/ApoB and RDW%) (Fig. S1). Notably, AUC-ROC values for the 6-protein panel were comparable to those obtained using the entire clinical-proteomic features set. Biologically, these proteins are involved in key atherosclerotic-related processes -such as platelet degranulation, fibrin clot formation, extracellular matrix organization, innate immune system, and plasma lipoprotein clearance. They have been linked to CVD related pathologies: aortic atherosclerosis, hyperlipidaemia, coronary artery disease, or vascular disease (Table S3).

**Fig. 5:**
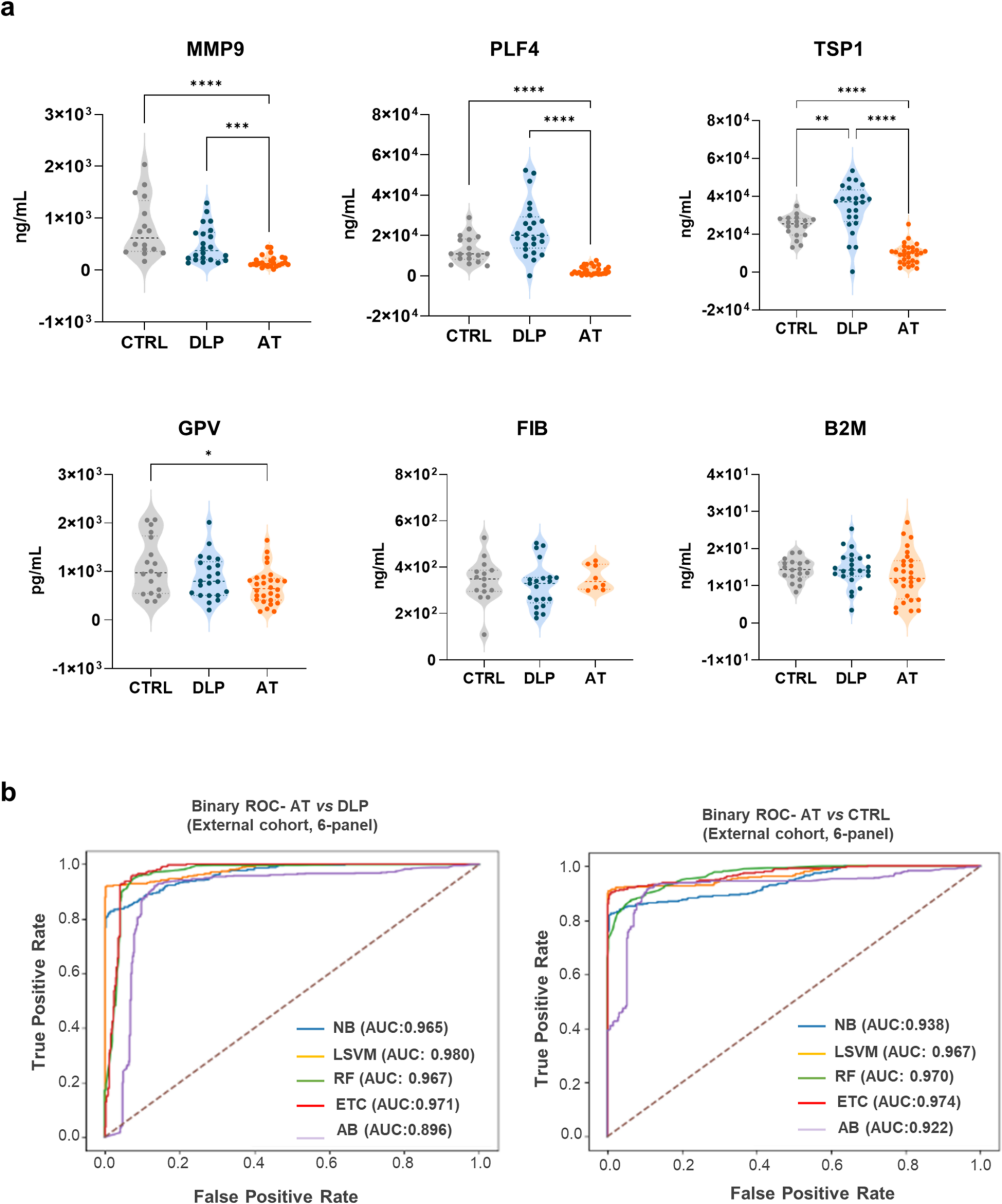
External validation of the 6-protein biomarker panel. **a** Representative violin plots (median, mix, max) of the changes detected for MMP9, PLF4, TSP1, B2M, FB (ng/ml) and GPV (pg/ml) in an external cohort of CTRL, DLP and AT stroke patients. Significant differences were calculated with Kruskal Wallis test and Dunn´s as post hoc test (*p-value < 0.05, **p-value < 0.01, ***p-value < 0.001). **b** Binary ROC curves and AUC values for the 6-protein panel calculated with the 5 MLCA models in AT compared to DLP and CTRL

Proteomic findings were first validated in the same discovery cohort (Fig. S2), using alternative tools available for each protein: GPV (ELISA), PLF4, MMP9, TSP1, B2M (immunocytometry) and FB (coagulometry). Furthermore, they were also tested in an independent external cohort (Fig. 5), which included older controls and equal numbers of male and female AT patients, to overcome the demographic differences of the initial set. In addition, the AT patients of the external cohort presented with stroke at the time of recruitment, representing a severe clinical complication of atherosclerosis. In line with the initial results, GPV, MMP9, PLF4 and TSP1 were significantly downregulated in the serum of AT patients compared to DLP and CTRL (Fig. 5a). Furthermore, B2M and FB showed upward trends, although such upregulation was not statistically significant. This may be attributed, at least for FB, to the low number of patients for whom this marker could be registered at hospitalization. Finally, an additional binary analysis of the 6-protein panel´s external values reported AUC-ROC values > 0.96 in four out of five classifiers for the AT *vs* DLP comparison. In the AT *vs* CTRL, the AUC-ROC ranged from 0.922 to 0.974 (Fig. 5b).

Overall, these results demonstrate that the 6-protein panel – whether used independently or integrated with the lipid features checked (ApoA1, ApoB, cLDL/ApoB ratio)- possesses high discriminative power to identify patients with severe AT, compared to both CTRL and DLP groups. Therefore, these findings support the 6-protein panel potential to serve as a serum biomarker for atherosclerotic severity stratification. Further analysis with a larger cohort will contribute to corroborate these findings.

### An integrated clinical-proteomic approach optimizes patient stratification

**Fig. 6:**
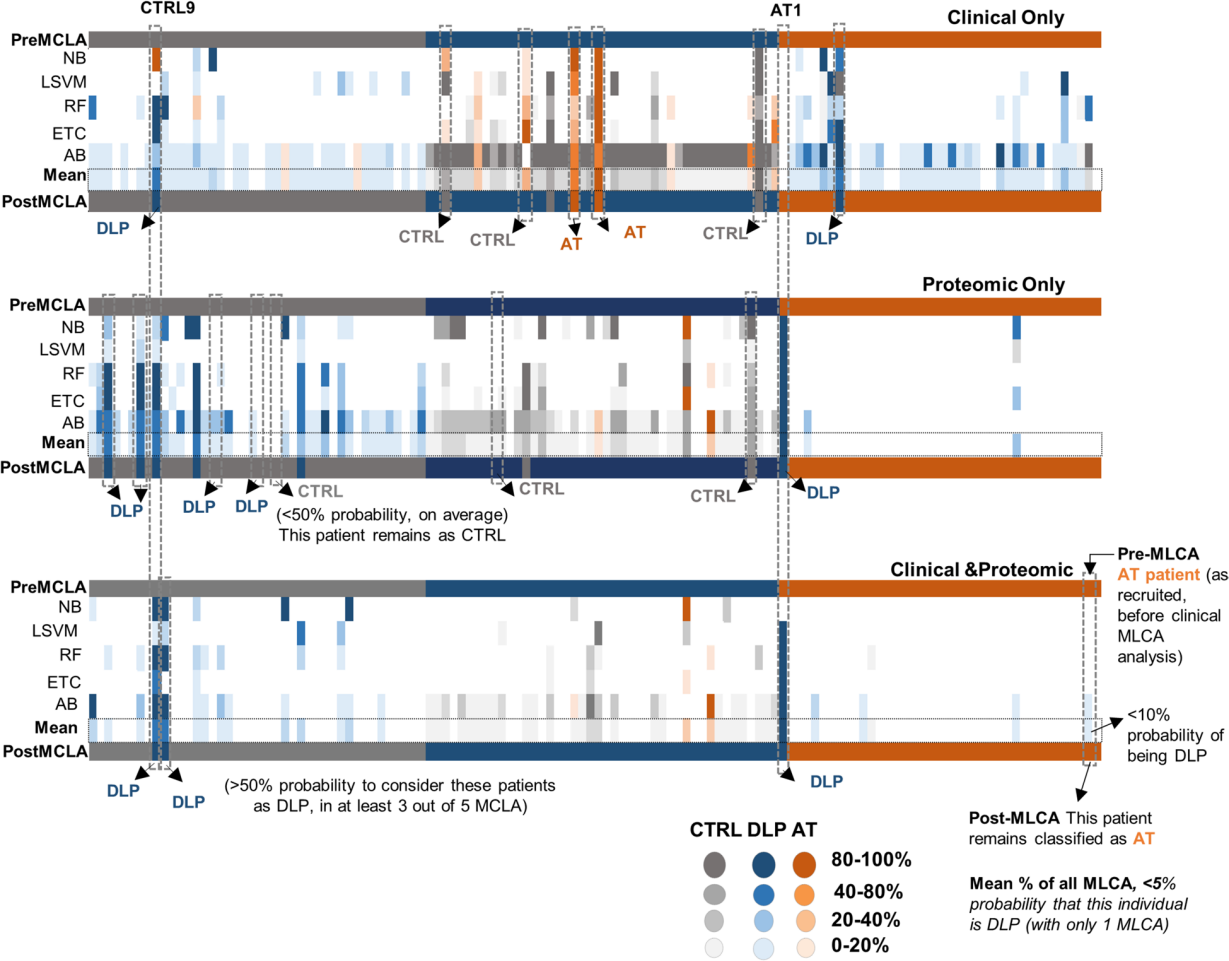
Schematic representation of patient stratification based on clinical, proteomic or clinical & proteomic MLCA analysis. CTRL individuals (grey), DLP (blue) or AT (orange) patients with carotid stenosis were initially grouped (pre-MLCA), based on clinical information (biochemical and hemogram values), plus doppler ultrasound data for AT patients. Five MLCA models analysed the clinical-only, proteomic-only, or the integrated clinical-proteomic data from all individuals. Doppler and therapeutic information were excluded from the stratification analysis. Individuals were reassigned to another category (post-MLCA) if at least 3 out of the 5 MLCA models showed a > 50% misclassification relative to pre-MLCA. Each column represents one patient; some examples have been highlighted to illustrate the reassignment logic. *NB: Naive Bayes, SVM: linear Support Vector Machine; RF: Random Forest, ETC: Extremely randomized trees classifier; AB: Ada Boost

After confirming the validity of the MLCA models, we further assessed their stratification power across the study groups (Fig. 6). The initial categorization (Pre-MLCA) relied mainly on clinical data (biochemical and hemogram values), plus doppler ultrasound results for AT patients. Subsequently, MLCA analysis was performed using the five models (NB, LSVM, RF, ETC, AB) with the clinical-only, proteomic-only and the combined datasets. Notably, doppler results and pharmacological treatment information were not considered for the stratification analysis. For each dataset, all five models reported a misclassification probability per individual: 0% indicated a correct assignment to the pre-MLCA group, while 100% indicated that the individual likely belonged to a different group. Consequently, individuals were reassigned to another category (post-MLCA) if at least 3 out of the 5 MLCA models yielded a misclassification probability exceeding 50%.

Among these models, AB demonstrated the lowest stratification specificity, reporting the highest misclassifications rates (ranging from 5 to 100%). Conversely, LSVM and ETC yielded lower number of misclassifications across the entire cohort, although this performance varied depending on the data source (clinical, proteomic or clinical&proteomic data). These findings suggest that relying on a single-model stratification approach carries a substantial risk of model-specific bias, which could lead to inaccurate assignments. However, our consensus-based strategy mitigated the individual model variance, ensuring that re-stratification decisions are driven by robust algorithmic consensus.

On the other hand, while most patients maintained their Pre- and Post-MLCA group assignments (CTRL, DLP or AT), the combined clinical-proteomic approach displayed the highest classification accuracy. Specifically, misclassifications rates dropped significantly from 11.17% (clinical-only) and 10.32% (proteomic-only) to 3.17% when clinical and proteomic data were combined.

Of note, the clinical-only analysis reported several misclassifications within the DLP group, identifying DLP individuals who were re-assigned (post-MLCA) as either controls or AT patients. Sometimes, the same individual was classified differently by different models, hindering a consensus stratification. In contrast, the proteomic-only and the combined clinical-proteomic approach effectively retained these individuals as “DLP”. Conversely, misclassifications in the proteomic-only analysis were predominantly found in the CTRL group (pre-MLCA), with several individuals reassigned as DLP (post-MLCA).

Remarkably, the combined clinical-proteomic stratification approach proved the most robust, identifying only 3 misclassified individuals: 2 CTRL and 1 AT (pre-MLCA). Notably, one control (CTRL9) was consistently reassigned as DLP across all three analyses (clinical, proteomic and clinical-proteomic tests). According to clinical records, CTRL9 had elevated cholesterol (225 mg/dl) and LDL (157 mg/dl) levels, which are classically considered borderline normal ranges. However, other parameters such as fibrinogen, not initially included in the pre-assignment but used for post-MLCA classification, was significantly elevated in CTRL9 (412 mg/dl) compared to other controls, supporting the “DLP” reclassification. Conversely, the AT1 patient, re-assigned as a DLP, reported very high lipid markers and elevated glucose levels compared to the rest of AT patients. According to clinical records, the AT1 patient was receiving a relatively low statin dose (20 mg/24 h) compared to the rest of AT cohort (60 mg on average). Thereby, such misclassification was indeed highlighting the uncontrolled, high-risk dyslipidemic profile of this AT patient.

Overall, the integrated clinical-proteomic approach offered the most accurate stratification, outperforming individual datasets analysis (Fig. 6). Our findings underscore the additive value of proteomics to standard clinical data for optimal patient stratification. Notably, the final stratification results (Post-MLCA), based solely on clinical and proteomic data, showed almost 97% concordance with the classification obtained after Doppler imaging. This suggest that the integrated method could serve as an effective initial screening tool, potentially precluding the need for universal imaging.

## Discussion

Despite the success of current therapies to reduce circulating levels of lipid particles involved in the formation of atherosclerotic plaques, the incidence of ACVD remains alarmingly high [31]. Consistent with this, the atherosclerotic group in our cohort exhibited the poorest clinical status, characterized by adverse CV risk factors, inflammatory and other relevant markers associated to ACVD, despite pharmacologically lowered lipid levels. These findings underscore the need for screening strategies incorporating additional markers beyond the standard lipid profile, to encompass a more accurate assessment of the atherosclerotic status.

In the current study, we applied a MLCA integrated approach combining clinical and proteomic data from CTRL, DLP patients with subclinical atherosclerosis and AT patients with advanced carotid stenosis. Consistent with recent research, proteomic data provided complementary information to the clinical results, offering a complete molecular picture of the individual´s status, and a more accurate patient stratification than the clinical parameters alone [22, 23]. Indeed, based on their proteomic profile, the AT group was clearly differentiated from DLP patients or healthy controls, whereas the latter two groups, although distinguishable, shared similar proteomic profiles. Ultimately, our findings demonstrate that the proteomic-driven approach was essential for identifying clinically relevant markers that standard assessments would have otherwise overlooked. For instance, APOB, APOA1 and B2M were identified by MS-proteomics as differential markers for AT patients. These changes were further validated by turbidimetry, and their values were subsequently integrated into the clinical dataset. Remarkably, all three proteins were finally identified as key predictive features by MLCA. Moreover, ApoB and cLDL/ApoB ratio, rather than LDL-c itself, were highlighted by MLCA as discriminating factors for AT. In agreement with our findings, various studies suggest that ApoB (LDL-c carrier) is a more accurate and reliable predictor of CVD risk than LDL-c itself. Furthermore, several organizations like ESC/EAs or AHA (PREVENT), now recommend including ApoB as an alternative to LDL-c, as the primary target for lipid-lowering therapy [20, 21].

Regarding the 5-MLCA averaged approach, this strategy mitigated the high error rates and false classifications produced by single classifiers. Indeed, the proposed strategy, where an individual´s reassignment (post-MLCA) was only accepted if at least 3 out of 5 models reached misclassification probabilities > 50%, proved optimal for rigorous re-stratification decision. Based on this combinatorial analysis, proteomic data yielded slightly more accurate group stratification than clinical information alone (10.32% *vs* 11.17% post-MLCA misclassifications respectively). Notably, the integrated clinical-proteomic approach achieved the highest discriminative power (3.17% misclassification post-MLCA), outperforming the individual analyses. Therefore, our model replicated approximately 97% of the initial cohort’s assignment by considering only serum proteomic and clinical features, without imaging or therapeutic information. Moreover, this strategy allowed the identification of patients with suboptimal therapeutic management and high-risk dyslipidemic profiles. Overall, these findings support the use of non-invasive proteomic blood tests as a viable alternative for early stratification; minimizing the need of relying on imaging data, at least in early stages of diagnosis.

The clinical-proteomic approach identified 16 discriminating features, including several markers associated with cholesterol and lipid transport (ApoAI, ApoB, LDL cholesterol to ApoB ratio), alongside RDW%, a standard measure of red blood cell volume heterogeneity, and 10 proteomic markers. Notably, elevated RDW% is associated with poorer outcomes and an increased risk of all-cause death among CVD [32, 33], suggesting that is inclusion into updated screenings could provide valuable prognostic insight.

Regarding the proteomic markers, we kept only those with AUC-ROC individual values > 0.7 for AT *vs* DLP and AT *vs* CTRL: B2M, GPV, MMP9, PLF4, TSP1 and Fibrinogen (in its 3 subunits, FIBA, FIBB, FIBG). The refined 6-protein panel showed AUC-ROC values > 0.97 across all MLCA models applied, matching the full 16-feature set and outperforming the individual proteins results. An external validation in an independent cohort of stroke-associated AT patients confirmed the significant downregulation of GPV, MMP9, PLF4 and TSP1 in the initial cohort, as well as the increase in B2M and FB levels, although this was only significant in AT patients with carotid stenosis but not in the stroke group. While future validation with larger datasets is required to confirm such tendencies, different studies have already demonstrated the correlation between B2M and FB upregulation and the risk of ischemic stroke [34, 35]. Remarkably, the 6-protein panel maintained high discriminating accuracy in the external cohort, with AUC-ROC values > 0.95 on average for all five classifiers for AT *vs* DLP and AT *vs* CTRL comparisons.

Overall, our findings corroborate the potential of the protein panel, whether alone or combined with lipid markers, as a signature biomarker of atherosclerosis severity. Indeed, the inclusion of these proteins into screening approaches would capture biological processes often missed by classical methods. Specifically, B2M and FB increase in AT patients reflects the systemic inflammatory response inherent to chronic atherosclerotic patients. B2M constitutes a robust marker of chronic inflammation [36], whose upregulation has been associated with asymptomatic carotid atherosclerosis [37, 38] and, moreover, with the risk of acute ischemic stroke [34] and with major adverse cardiovascular events [39–41]. Likewise, FB is recognized as an acute-phase reactant whose levels increase during inflammation [42], accelerating the progression of atherosclerosis [43]. Furthermore, FB plays a major role in the clotting process [44], increasing the risk of thrombotic events [35]. Consequently, elevated FB is currently considered both a marker and mediator of severe atherosclerosis and an independent predictor of atherothrombotic stroke [45–47]. Therefore, FB has demonstrated a greater prognostic impact in atherosclerotic patients than increased serum cholesterol concentrations alone [42].

On the other hand, the downregulation of TSP1, PLF4, GPV and MMP9 in the AT serum, may reflect their acute accumulation within the atherosclerotic area (Fig. S3). For instance, GPV is essential for anchoring platelets to damaged vessel walls via the GPIb-V-IX receptor complex and its interaction with ECM proteins, such as von Willebrand Factor, during the early phases of AP formation [48]. Similarly, TSP1 and PLF4 participate in various processes such as platelet aggregation, matrix production and organization and thrombus adhesion to injured blood vessels [49–53]. Specifically, TSP1 accumulation seen within calcified atherosclerotic lesions [54], directly mirrors the serum downregulation observed in our AT patients. Consistent with this, reduced TSP1 serum levels have been reported in patients with chronic heart failure [55, 56], abdominal aortic aneurysm [57] or hypertension compared to healthy controls [58], supporting its potential role as CVD marker. Likewise, the presence of PLF4 (also known as CXCL4) in atherosclerotic areas correlates with the severity of the atherosclerotic lesion [59]. As a pro-inflammatory chemokine, PLF4 recruits inflammatory cells to the site of injury, stimulating the release of matrix metalloproteinases like MMP9 [60]. Remarkably, MMP9 itself accumulates in vulnerable plaque regions [61, 62], where it promotes ECM degradation [61] and inflammatory cell infiltration through the lipid necrotic core [63], thereby driving persistent inflammation [64, 65].

Beyond these roles, these proteins may contribute to plaque instability through a complex modulation of the angiogenic balance inherent to plaque instability. On one hand, accumulation of the pro-angiogenic factor MMP9 [66, 67], might promote the growth of fragile, immature vessels [68], contributing to intra-plaque hemorrhages (IPH) [69] and the progression toward vulnerable plaques prone to rupture [70–72]. Conversely, PLF4 and TSP1 accumulation (natural angiogenic inhibitors) [71], within the plaque may compromise lesion stability, by blocking the compensatory neovascularization required by the hypoxic necrotic core [73]. Therefore, dysregulated angiogenesis and IPH likely do not trigger plaque rupture directly, but rather promote certain events (e.g., necrotic core expansion, increased inflammation and fibrous cap thinning) that ultimately lead to plaque disruption [65, 74].

Taken together, while further validation of this hypothesis is required, the systemic depletion seen of these platelet-related proteins in AT patients likely represents their sequestration within the atherosclerotic lesion. Such accumulation contributes, among others, to the development of a vulnerable necrotic core and intraluminal thrombus (ILT) formation [61, 75–77], accelerating the transition to advanced stages of atherosclerosis [78].

Our study has certain limitations that must be acknowledged. First, the current cohort sample size was relatively small; thus, validation in larger, multi-centre cohorts is necessary to confirm the robustness of the identified biomarkers and the potential clinical translation of our MLCA approach. Second, we cannot rule out the impact of pharmacological treatments predominantly administered in the AT group, like statins, antiplatelet agents, and antidiabetic therapies, which may modulate some of the highlighted markers [79, 80]. For instance, MMP9 levels can be downregulated by aspirin [81], statins [82–85] or even antidiabetic agents [86–88], while statins may also inhibit TSP1 activity [89] and aspirin appears to increase GPV shedding [90]. Therefore, further longitudinal analyses with pre- and post-treatment cohorts are required to validate our findings and isolate potential therapeutic effects.

We also acknowledge the heterogeneity of our initial cohort, specifically the differences in age and sex distribution between healthy controls and the patient groups. However, the inclusion of the second cohort in the validation phase somehow mitigated these biases. Notably, while the clinical-only approach identified age as discriminating feature, the integrated clinical-proteomic model achieved high accuracy classification (97%) without considering age or sex variables. Ultimately, the goal is to develop an optimal stratification model to distinguish patients with severe atherosclerosis from those with subclinical dyslipidemia, and thereby predict future risk. Therefore, the use of a younger, healthy baseline remains clinically relevant to identify molecular deviations before the onset of advanced, age-associated comorbidities.

Despite these limitations, the integration of proteomic and clinical data proved highly effective, outperforming individual datasets in stratifying patients. Furthermore, our consensus ML-strategy, requiring agreement from at least 3 out of 5 models for reassignment, significantly mitigated the risk of overfitting and false classifications often associated with small datasets or single-classifier approaches. Notably, our consensus ML model achieved high accuracy levels despite significant differences in age or pharmacological treatments, which strongly suggests the identified biomarker panel as a robust, independent signature of disease severity, rather than a reflection of demographic confounders. Moreover, stratification was achieved without the need for imaging, supporting the use of proteomic serum biomarkers for initial screening. Finally, the stratification process highlighted a subset of high-risk, therapeutically uncontrolled dyslipidemic patients within the AT cohort.

Overall, the protein panel identified underscores the complexity of the atherosclerotic process and the limitations of relying solely on lipid markers for risk stratification. For instance, our data suggest that soluble platelet biomarkers may offer superior predictive value for thrombotic events than conventional platelet tests, while also serving as an indicator of IPH [91, 92]. Furthermore, angiogenic modulators might contribute to mitigate IPH and reduce plaque vulnerability and rupture. Therefore, a multi-target approach may constitute a better strategy to treat atherosclerosis, warranting further investigation in this direction.

## Materials and methods

### Study population

Patients and healthy controls (18–75 years old) were enrolled (n:181 in total) between years 2018–2022 at the Joaquin Pece primary health care center, the University Hospital Puerta del Mar (HUPM) and University Hospital of Jerez de la Frontera, Cádiz, Spain. Due to COVID-19 pandemic, individuals recruited between February-April 2020 were screened via serology (for specific IgG and IgM antibodies), and with nasopharyngeal qPCR tests against SARS-CoV-2. Individuals testing COVID-19 positive were excluded from the study. Briefly, participants accomplishing inclusion criteria were classified in three different groups: AT patients (AT, n:60), diagnosed with > 70% of carotid artery stenosis in doppler ultrasound; DLP (n:55), diagnosed based on biochemical parameters, as described [93]: HDLc < 40 mg/dL (men); < 50 mg/dL (women) and LDLc > 160 mg/dL; and healthy controls (CTRL, n:66), with HDL > 40 mg/dL (men); > 50 mg/dL (women); and LDL < 160 mg/dL.

### Blood sample collection and clinical analyses

Blood samples were collected after overnight fasting, using SST II Advance, K2E (EDTA) and Citrate/CTAD tubes (BD Vacutainer, Plymouth, United Kingdom). Biochemical parameters were obtained by photometric assays, using a Hitachi Cobas c702 (Roche Diagnostics, Rotkreuz, Suiza) and Alinity c (Abbott, IL, USA) instruments. Hemogram parameters were obtained in a Sysmex analyzer (Sysmex, Kobe, Japan).

For the proteomic analysis, serum samples were also collected, with an extra tube of SST II Advance. Briefly, after clot formation, samples were centrifuged 10 min at 2000 g, at 4 °C. All serum samples were aliquoted and stored at −80 °C for further analysis.

### Proteomic analysis

In total, 10 $\mu$L of serum samples (~ 600 $\mu$g) were denatured, reduced and alkylated with 100 $\mu$L 1% SDS, 5 mM TCEP, 10 mM CAA, 100 mM Tris (pH 8,5) and a tablet of Complete Mini, EDTA-free Protease inhibitor cocktail, during 10 min at 95ºC and 1400 rpm. Next, serum samples were digested overnight using the protein aggregation capture (PAC) protocol, as previously described [94, 95]. Briefly, protein samples, in a final concentration of 70% acetonitrile, were incubated with magnetic Amine beads (ReSyn Biosciences) in a protein/bead ratio of 1:3. PAC was carried out in two steps of 1 min, mixing thoroughly, followed by 3 consecutive washes with 95% acetonitrile and twice with 70% ethanol twice, 5 min each, without releasing the beads from the magnet. On-bead digestion was set up to 12 h at 37ºC, in agitation, using 1:50 Trypsin/LysC Mix (Promega) in 50 mM ammonium bicarbonate solution. The digestion was quenched with 1% trifluoroacetic acid. Finally, peptide samples (20 µl) were loaded directly onto Evotips (Evosep) for MS analysis.

### LC–MS/MS

Peptide samples were analyzed using an Evosep One system connected online to the Orbitrap Exploris 480 MS (Thermo Fisher Scientific, Bremen, Germany). Chromatographic separation was performed on a 15 cm capillary column (75 μm i.d.) with 1.9 μm Reprosil-Pur C18 beads (Acclaim™ PepMap™ 100 C18, Thermo Scientific). The column temperature was set at 60 °C with an integrated column oven (PRSO-V1, Sonation, Biberach, Germany). Peptides were separated at a flow rate of 30 μl/min with buffer A (0.01% formic acid) and buffer B (100% acetonitrile). The mass spectrometer was operated in independent data acquisition (DIA) mode. Full MS scans were acquired at 120,000 resolution, with a full scan range of 350 to 1400 m/z and the AGC target of 300%. Fragment ion scans were recorded at a fixed resolution of 30,000 and with a maximum IT of 54 ms and a normalized AGC target of 1000%. 26 windows of 25.9 m/z scanning from 361 to 1033 m/z were used. The isolated ions were fragmented using HCD with 27% NCE.

### Raw data processing and data analysis

Raw files were processed using Spectronaut (v15.4) with a library-free approach (directDIA) using human database (Uniprot reference proteome 2020 release, 20,600 entries). Search parameters included cysteine carbamylation (fixed modification), and methionine oxidation and N-termini acetylation (variable modifications). Enzyme was established to Trypsin, with a maximum of one tryptic missed-cleavages allowed. Minimal peptide length was set to 7 amino acids. Results were filtered at 1% FDR (peptide and protein level), considering only proteins with at least two peptides identified for further analysis. LFQ was done with match between runs (match window of 0.7 min and alignment window of 20 min).

Data processing was conducted in Perseus (v1.6.15.0) and R. Briefly, data were log2 transformed and filtered to retain proteins identified with valid values in at least 70% of the total samples analyzed. Missing values were imputed from normal distribution, followed by quantile normalization. Differentially expressed proteins were identified using a two-sample student’s *T*-test with a permutation-based FDR, considering those with q-value < 0.05.

Finally, functional enrichment analysis of the differential proteins identified was performed using String (www.string-db.org), against the human plasma background dataset (www.proteinatlas.org). Reactome (Home—Reactome Pathway Database), and Ingenuity® Pathway Analysis (IPA®, QIAGEN Redwood City) software were used to identify potential connections between the altered proteins and atherosclerosis related diseases.

### Turbidimetric assays

To validate the proteomic results, the levels of several proteins already associated with CVDs (A1AG1, APOA1, APOB, APOE and B2M) were further analyzed, using the quantitative turbidimetric test (TURBI, Spinreact, Gerona, Spain) with the BS-200E analyzer (Mindray, Shenzhen, China).

### Validation of the protein biomarker panel

The proteomic variations found for the biomarkers TSP1, PF4, MMP9, FB and B2M, were first validated with a subset of individuals from the initial cohort (CTRL, n:26; DLP, n:27; AT, n:27), followed by a second validation with an external independent cohort, which included CTRL (n:19) and DLP (n:24) patients from Joaquin Pece care center, and AT patients (n:30) recruited at the Virgen Macarena University Hospital in Seville. AT patients presented at recruitment with symptoms suggestive of acute stroke, and received a confirmed diagnosis by neuroimaging, based on World Stroke Organization (WSO) criteria [96].

In both cases, the levels of TSP1, PF4, MMP9 and B2M were measured by flow cytometry, at RayBiotech Life´s facilities, using a custom-made quantitative multiplexed bead-based quantitative antigen array (RayPlex® Custom Antigen Array). Fibrinogen changes were validated by coagulometry, in an ACL Top (Werfen, Barcelona, Spain), while GPV serum levels were tested using the Human GP5 ELISA Kit (EH2178, Whuan Fine Biotech Co., Wuhan, China), following manufacturer’s instructions. Results were obtained at 450 nm in the microplate reader Fluostar Omega (BMG Labtech, Ortenberg, Germany).

### Statistical analysis

Statistical analysis of baseline characteristics was performed using SPSS Statistics software v.20.0.0 (Chicago, IL, USA). Data were presented as absolute numbers and percentages, or as the mean values ± standard deviation (mean ± sd) for categorial or continuous variables respectively. Kolmogorov–Smirnov or Shapiro–Wilk normality tests were applied, and independent sample Chi square test, Kruskal–Wallis test and Dunn´s as post hoc test were conducted accordingly. Null hypothesis was rejected if p value < 0.05.

Graphs for individual protein changes (LFQ intensities or Turbidimetric results) were obtained with GraphPad Prism 7 software (Boston, MA, USA). Outliers were identified via the ROUT method, followed by One-t-test ANOVA analysis with the Tukey post hoc test. Also, ROC curves were plotted and AUC values were calculated for individual proteins, using the Youden index for cut off value. Finally, Spearman multivariate correlations analysis was run with RStudio software, using the *stats* and *Hmisc* packages. Correlations with |R|> 0.6 or |R|< −0.6 were selected.

### Machine learning algorithms

Five supervised MLCA, were evaluated for patient stratification: NB [26], SVM [27], RF [28], ETC [29] and AB [30]. Missing values were imputed using the k-Nearest Neighbours (kNN) algorithm (k = 5, weighted Euclidean distance) [97]. All analyses were performed in Python (v3.6) using the scikit-learn library (https://scikit-learn.org/stable/) [98].

A rigorous feature selection pipeline was implemented to minimize overfitting and information leakage. The procedure combined a 3-repeated, fivefold stratified cross-validation scheme, with internal bootstrap resampling. Within each iteration, clinical and proteomic variables were selected from standardized training data, using Recursive Feature Elimination (RFE) [99] or univariate F-tests as fallback. Within each fold, variables retained in at least 60% of the bootstrap runs were recorded. The final consensus panel retained features appearing in ≥ 20% of validation folds. To prevent redundancy, highly correlated predictors (Spearman’s ρ ≥ 0.85) were grouped, and only the most frequently selected representative of each correlated cluster was retained.

Next, model training and evaluation were conducted via 20-repeated stratified threefold cross-validation, reporting standard performance metrics: accuracy, precision, recall, F1-score, AUC-ROC values and 95% CI. Model interpretability and feature relevance were evaluated using either native feature importance (RF, ETC, AB) or absolute model coefficients (LSVM), and SHapley Additive exPlanations (SHAP) values. A consensus ranking and heatmap were generated to highlight variables recurrently contributing to classification performance, and correlations among top-ranked predictors were explored to detect redundancy or synergistic effects (Fig. S4-S6).

Finally, the rate of individual´s misclassifications was quantified. Thus, individuals were flagged as inconsistently classified when appeared misclassified > 50% in at least 3 out of the 5 MLCA models, warranting further examination. Additional details for methods and materials are provided in the Supplementary Methods and Materials.

## Supplementary Information

Additional file 1. Supplementary Materials and Figures.

Additional file 2. Supplementary Tables.

## Data Availability

The data underlying this article are available within the article and its online supplementary material. Additional information will be provided by the corresponding authors upon reasonable request. In addition, mass spectrometry proteomics data have been deposited to the ProteomeXchange Consortium via the PRIDE partner repository [100] with the data set identifier PXD049351.

## Abbreviations

AB: AdaBoost
ACVD: Atherosclerotic cardiovascular diseases
AMI: Myocardial infarction
AP: Atherosclerotic plaques
AT: Atherosclerosis
CTRL: Control
CV: Cardiovascular
DLP: Dyslipidemic
ETC: Extremely Randomized Trees Classifier
GP: Gaussian Process
HDLc: High density lipoprotein-cholesterol
IPH: Intra-plaque hemorrhages
LC–MS/MS: Liquid chromatography-Tandem Mass spectrometry
LDLc: Low density lipoprotein-cholesterol
LFQ: Label free quantification
MLCA: Machine Learning classification algorithms
NB: Naive Bayes
RF: Random Forest
SVM: Linear Support Vector Machine
