# Supplementary material for: Machine learning integrated clinical-proteomics data identifies a 6-protein panel signature for atherosclerotic severity and enhanced patient stratification
Source: Mol Biomed. 2026 Apr 10;7:49. doi: 10.1186/s43556-026-00438-z (PMC13069073; doi:10.1186/s43556-026-00438-z)
Supplement: Supplementary file 1 — Additional file 1. Supplementary Materials and Figures. [file 43556_2026_438_MOESM1_ESM.docx]

**TITLE: Machine learning integrated clinical-proteomics data identifies a 6-protein panel signature for atherosclerotic severity and enhanced patient stratification**

**AUTHORS:** Mª Jesús Extremera-García^1,2^, Marta Rojas-Torres^1^, Blanca Priego-Torres^3*^, Lucía Beltrán-Camacho^1^, Sara Eslava-Alcón^1^, Francisco Rodríguez-Martín^4^, Josefa Benítez-Camacho^1^, Antonio Ballesteros-Ribelles^1^, Ana Martínez del Val^5,6^, Jesper Olsen^6^, Eva Lozano-Loaiza^7^, Mª Ángela González-García^8^, Daniel Sanchez-Morillo^3^, Alejandro Fernández-Vega^9^, Joan Montaner^9^, Esther Doiz^2,10^, Manuel Rodriguez-Piñero^2,10^, Mª Carmen Durán-Ruiz^1*^

**AFFILIATIONS:**

^1^Biomedicine, Biotechnology and Public Health Department, Cádiz University, Cádiz, Spain//Biomedical Research and Innovation Institute of Cadiz (INiBICA), Cadiz, Spain

^2^UGC Laboratory Medicine, University Hospital Puerta del Mar, Cádiz, Spain

^3^Automation Engineering, Electronics and Computer Architecture and Networks Department, University of Cádiz, Cádiz, Spain//Biomedical Research and Innovation Institute of Cadiz (INiBICA), Cadiz, Spain

^4^Institute of Biomedicine of Seville (IBIS), Virgen del Rocio University Hospital//CSIC/ Dpt. Cell Biology, Faculty of Biology, University of Seville, Seville, Spain.

^5^Current affiliation. National Center of Cardiovascular research Carlos III (CNIC), Madrid, Spain

^6^Novo Nordisk Foundation Center for Protein Research, University of Copenhagen, Copenhagen, Denmark

^7^Internal Medicine Unit, La Línea Hospital, La Línea de la Concepción, Cádiz, Spain

^8^UGC Laboratory Medicine, University Hospital of Jerez de la Frontera, Cádiz, Spain

^9^Neurovascular Research Group, Institute de Biomedicine of Seville, IBiS/Virgen Macarena University Hospital/CSIC/University of Seville, Seville, Spain

^10^Angiology and Vascular Surgery Unit, University Hospital Puerta del Mar, Cádiz, Spain// Biomedical Research and Innovation Institute of Cadiz (INiBICA), Cadiz, Spain

***Corresponding authors:**

**Blanca Priego-Torres,** [blanca.priego@uca.es](mailto:blanca.priego@uca.es)

Automation Engineering, Electronics and Computer Architecture and Networks Department, University of Cádiz, Cádiz, Spain

**Mª Carmen Durán-Ruiz, PhD**; maricarmen.duran@gm.uca.es

Biomedicine, Biotechnology and Public Health Department, Faculty of Medicine. Cádiz University.

Plaza Falla, nº9. Cadiz, Spain. CP 11003.

ORCID ID: 0000-0002-1700-0141

**SUPPLEMENTARY FIGURES**

**Supplementary Figure 1.** Representative nomograms, comparing the results for the “proteomic” selected features (a), and the “lipid-related” selected features (b). The proteomic panel reached higher AUC-ROC values than the individual lipid markers alone, fo both, AT vs DLP and AT vs CTRL comparisons.

1. **Features: TSP1, GPV, PLF4, MMP9, FIBBA, FIBB, FIBG, B2MG**


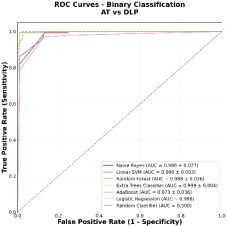

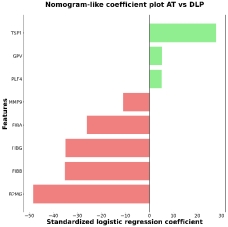

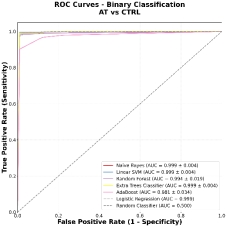

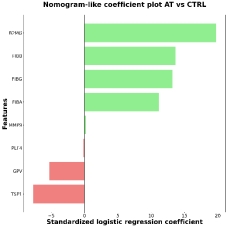

1.
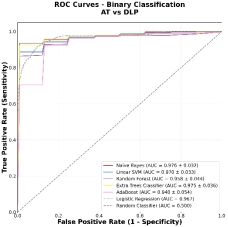

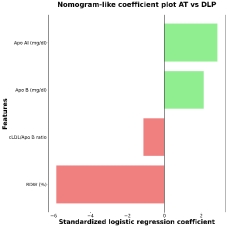

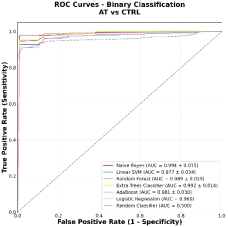

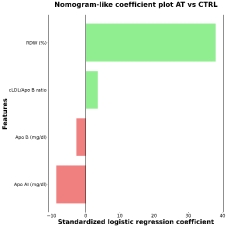

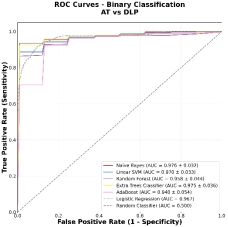

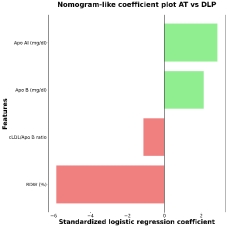
**Features: Apo AI (mg/dl), cLDL/ApoB ratio, ApoB (mg/dl), RDW%**

**Features: "Apo AI (mg/dl)", "cLDL/Apo B ratio", "Apo B (mg/dl)", "RDW (%)"**

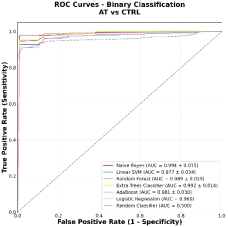

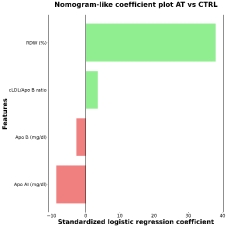

**Supplementary Figure 2. a)** Graphical representation of the proteomic changes seen for the proteins highlighted and b) the results obtained for the same proteins with the alternative tools available: GPV (ELISA), PLF4, MMP9, TSP1, B2M (flow cytometry), and FB (Coagulometry), in the same cohort.

**Supplementary Figure 3: Schematic representation of the proposed model linking systemic protein downregulation to local plaque pathology.** Illustration of the inverse relationship between circulating serum levels and local plaque abundance. The observed systemic downregulation of TSP1, PLF4, MMP9, GPV, and FB in Atherothrombotic (AT) patients is hypothesized to result from their active sequestration within the fibrous cap and necrotic core of the atherosclerotic plaque. This local accumulation is associated with platelet activation, inflammatory cell recruitment, and uncontrolled angiogenesis, potentially contributing to leaky vessel formation and intraplaque hemorrhage (IPH). These pathological events collectively drive plaque progression toward instability and thrombotic events. Image adapted from Khraishah et al [1] *)* under CC BY license.


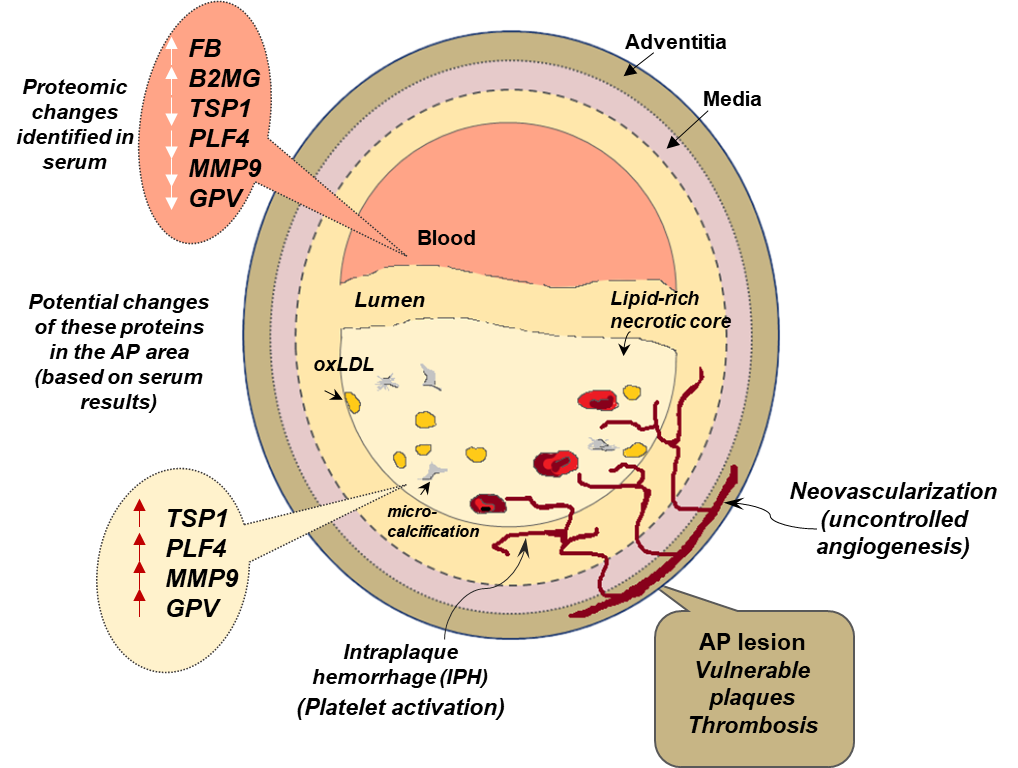


**Reference:**

Khraishah, H. and F.A. Jaffer, *Intravascular Molecular Imaging: Near-Infrared Fluorescence as a New Frontier.* Front Cardiovasc Med, 2020. **7**: p. 587100.

**Supplementary Figure S4. Consensus heatmap of feature importance.** Normalized feature importance scores across all classifiers were aggregated to assess consistency in variable contribution. The heatmap shows that *fibrinogen subunits* and *Apo B* maintained high normalized importance across nearly all models, whereas other variables such as *COR1A* and *MMP9* exhibited model-specific patterns.


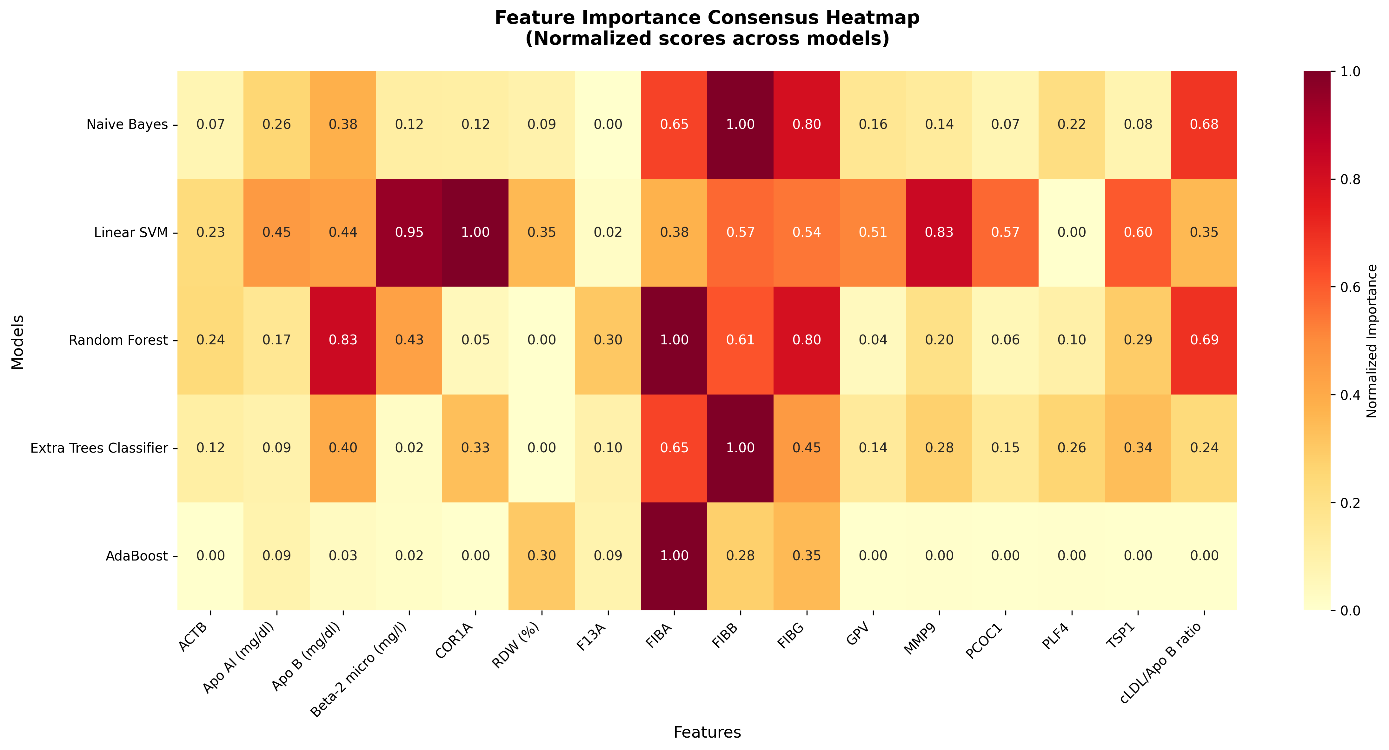


**Supplementary Figure S5. Correlation matrix of top consensus features.**
Pairwise correlations among the eight most influential variables were examined to explore redundancy and interactions. The three *fibrinogen subunits* were highly correlated (ρ ≈ 0.98–0.99), as expected from their shared biological role, whereas moderate inverse correlations were observed between *fibrinogen chains* and *MMP9* or *COR1A*, reflecting distinct but complementary biological processes.


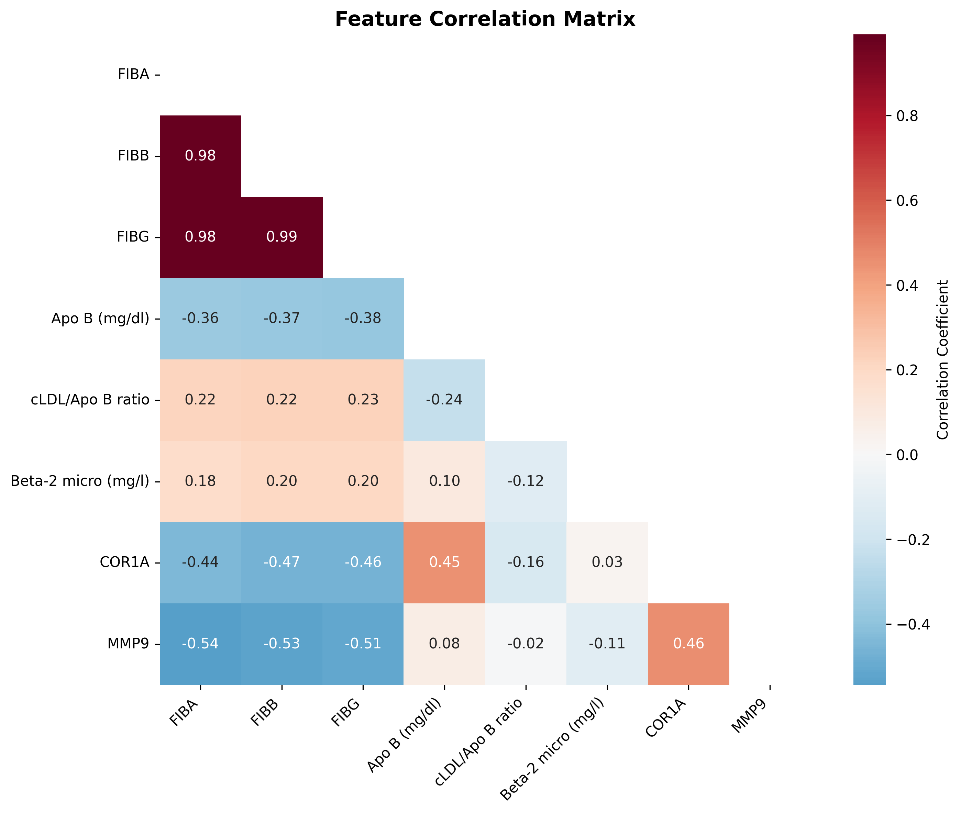


**Supplementary Figure S6. SHAP and Meta-Waterfall Plots for Model Interpretability.** SHAP waterfall plots illustrate how individual features contribute to a model’s prediction for each patient, showing the additive progression from the baseline expectation to the final output. Each horizontal bar represents a feature, ordered by the magnitude of its contribution, indicating whether it increases or decreases the prediction score. The meta-waterfall plots integrate results from multiple classifiers, displaying the average SHAP contribution of each feature across models. Color saturation reflects the level of inter-model agreement, while the percentage shown on each bar denotes the proportion of classifiers that share the same direction (sign) of contribution. Together, these visualizations highlight the most influential and consistently predictive features at both the model and ensemble levels.


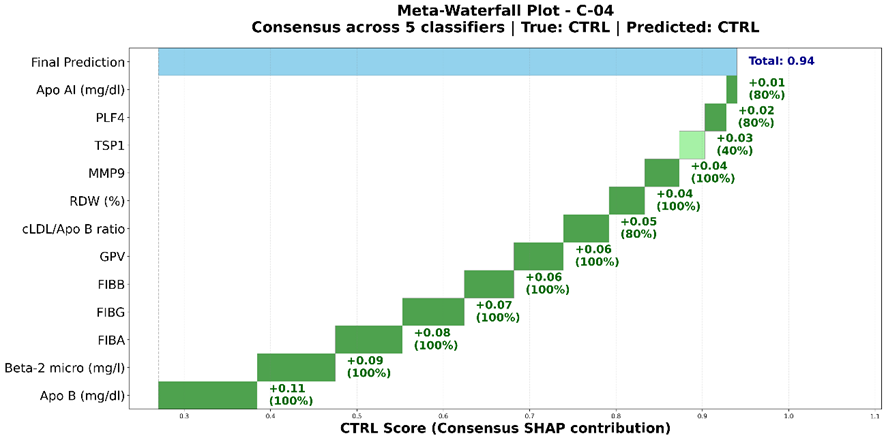

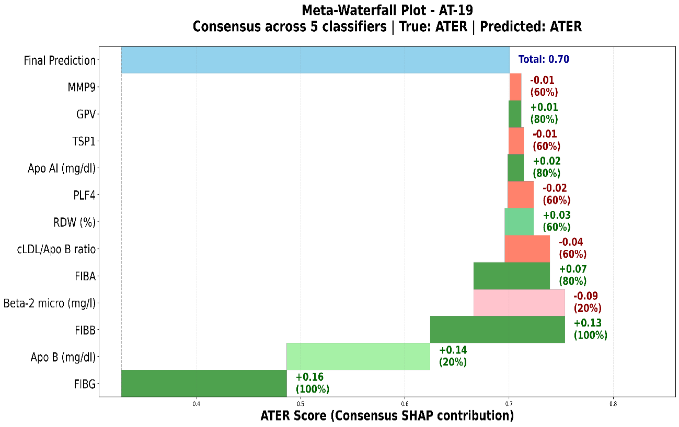


**Meta-Waterfall Plots**

**Final prediction**

Apo AI

PLF4

TSP1

MMP9

RDW%

cLDL/ApoB ratio

GPV

FIBB

FIBG

FIBA

B2M

ApoB

**CTRL04 (Pre-MCLA). Predicted: CTRL**


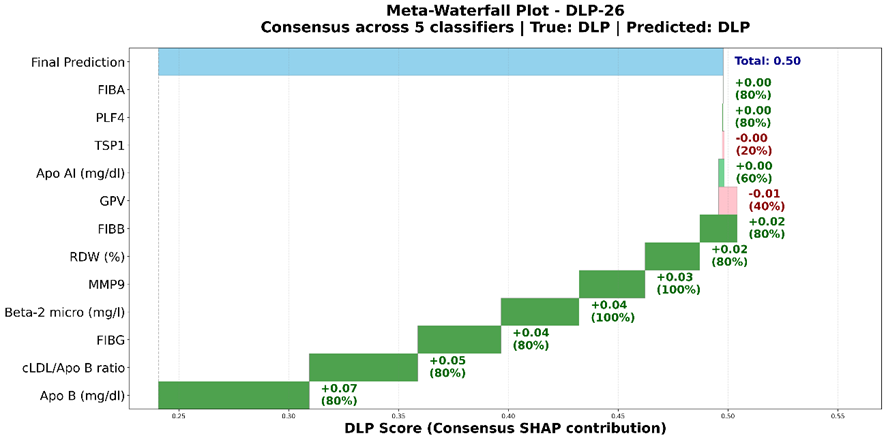


**DLP26 (PreMLCA). Predicted: DLP**

FIBA

PLF4

TSP1

APOA1

RDW%

cLDL/ApoB ratio

GPV

FIBB

FIBG

MMP9

B2M

ApoB

**Final prediction**


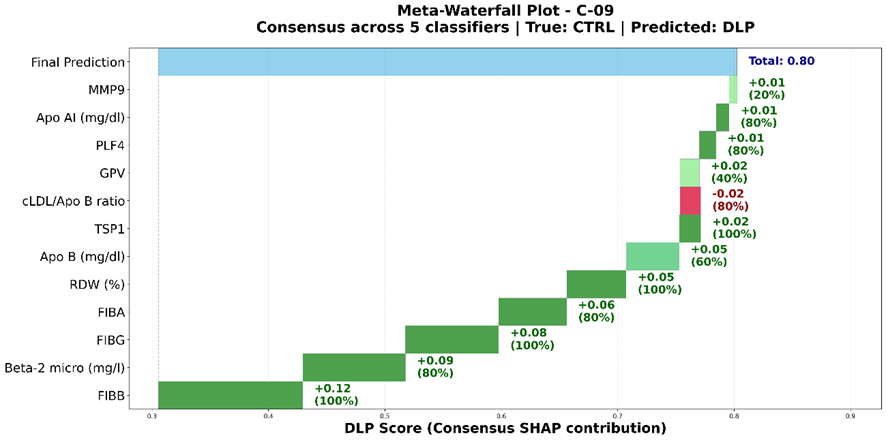


**CTRL09 (Pre-MCLA). Predicted: DLP**

Apo AI

PLF4

TSP1

MMP9

RDW%

cLDL/ApoB ratio

GPV

FIBB

FIBG

FIBA

B2M

ApoB

**Final prediction**


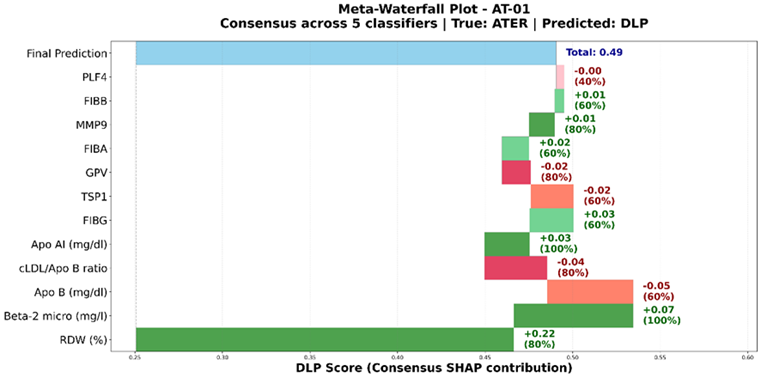


FIBB

MMP9

GPV

PLF4

RDW%

cLDL/ApoB ratio

FIBA

APOB

FIBG

APOA1

B2MG

TSP1

**AT1 (PreMLCA). Predicted DLP**

**Final prediction**

Apo AI

PLF4

TSP1

MMP9

RDW%

cLDL/ApoB ratio

GPV

FIBG

FIBA

B2M

ApoB

FIBB

**AT19 (Pre-MCLA). Predicted: AT**
